# Multi-omics analysis of the oncogenic role of optic *atrophy 1* in human cancer

**DOI:** 10.18632/aging.205214

**Published:** 2023-11-16

**Authors:** Ziyi Wu, Nuo Xu, Guoqing Li, Wen Yang, Chen Zhang, Hua Zhong, Gen Wu, Fei Chen, Dianqing Li

**Affiliations:** 1 Department of Orthopedics, The Second Xiangya Hospital of Central South University, Changsha, Hunan 410011, China; 2 Department of Spine Surgery, The Second Xiangya Hospital, Central South University, Changsha, Hunan 410011, China; 3 Xiangya School of Medicine, Central South University, Changsha, Hunan 410013, China; 4 The Department of Network Center, Hainan Normal University, Haikou, Hainan 571158, China; 5 Department of Emergency, The Fourth People’s Hospital of Zigong, Zigong, Sichuan 643000, China; 6 Department of Orthopedics, The Fifth Affiliated Hospital, Southern Medical University, Guangzhou, Guangdong 510900, China

**Keywords:** *OPA1*, pan-cancer, prognosis, immune infiltration

## Abstract

Objective: To investigate the prognostic significance of *optic atrophy 1* (*OPA1*) in pan-cancer and analyze the relationship between *OPA1* and immune infiltration in cancer.

Results: *OPA1* exhibited high expression levels or mutations in various types of tumor cells, and its expression levels were significantly correlated with the survival rate of tumor patients. In different tumor tissues, there was a notable positive correlation between *OPA1* expression levels and the infiltration of cancer-associated fibroblasts in the immune microenvironment. Additionally, *OPA1* and its related genes were found to be involved in several crucial biological processes, including protein phosphorylation, protein import into the nucleus, and protein binding.

Conclusion: *OPA1* is highly expressed or mutated in numerous tumors and is strongly associated with protein phosphorylation, patient prognosis, and immune cell infiltration. *OPA1* holds promise as a novel prognostic marker with potential clinical utility across various tumor types.

Methods: We examined *OPA1* expression in pan-cancer at both the gene and protein levels using various databases, including Tumor Immune Estimation Resource 2.0 (TIMER 2.0), Gene Expression Profiling Interactive Analysis (GEPIA2), UALCAN, and The Human Protein Atlas (HPA). We utilized the Kaplan-Meier plotter and GEPIA datasets to analyze the relationship between *OPA1* expression levels and patient prognosis. Through the cBioPortal database, we detected *OPA1* mutations in tumors and examined their relationship with patient prognosis. We employed the TIMER 2.0 database to explore the correlation between *OPA1* expression levels in tumor tissue and the infiltration of cancer-associated fibroblasts in the immune microenvironment. Furthermore, we conducted a gene search associated with *OPA1* and performed enrichment analysis to identify the main signaling pathways and biological processes linked to them.

## INTRODUCTION

Tumor development is a complex process, involving numerous factors such as altered gene expression, gene mutations, changes in protein structure, and changes in the tumor immune microenvironment [1–3]. Cancer poses a significant danger to human life and health and has a significant impact on socio-economic progress. Many tumors are also frequently encountered in clinical work. Breast cancer, head and neck squamous cell carcinoma, sarcoma, and skin cutaneous melanoma are all common tumors in plastic and aesthetic (burn) surgery [4–7]. As of now, the etiology and mechanisms of tumors have not been fully understood [8]. Therefore, the identification of those genes that were highly expressed in various tumor tissues and the assessment of their effects on the survival of tumor patients is important for discovering new prognostic markers for tumors as well as providing clinicians with new therapeutic ideas [9–12]. In recent years, many researchers have focused on mitochondria and have found structural or functional alterations in mitochondria in patients with certain diseases. This suggests that there may be some connection between the structure and function of mitochondria and the development of disease [13–15]. It has been found that in some common diseases including neurodegenerative diseases and cancer, pathological alterations in the cellular environment can cause changes in the function or activity of many proteins associated with mitochondrial fusion or cleavage. These changes will directly affect mitochondrial fusion or cleavage, resulting in altered mitochondrial dynamics [16].

The *OPA1* gene is located at 3q28 and is over 100 kb in length with 31 exons [17]. Previous studies have revealed the presence of an *OPA1* protein encoded by the *OPA1* gene in mitochondria, which is necessary for angiogenesis. On the other hand, angiogenesis is necessary for tumor growth and metastasis [18]. In addition, mitochondria are key organelles required to maintain normal endocrine function, providing energy for hormone production and transport [19]. The endocrine level is related to the occurrence, development, and prognosis of various tumors. Some researchers used endocrine disruptors to affect the function of the endocrine system and interfere with hormone action, which could increase the risk of tumors including reproductive damage, metabolic disorder, breast cancer, and prostate cancer [20]. In animal models, endocrine disruptors have carcinogenic effects on endocrine response tissues such as the breast, prostate, testis, ovary, and thyroid [21–23]. Therefore, it is important to study the factors affecting mitochondrial function and its influence on the endocrine level to find out the occurrence and development of some cancers and find new prevention and treatment measures.

Mitochondria are highly dynamic cellular organelles, exhibiting diverse morphologies such as small spheres, short or long tubules, and interconnected structures [24]. These shapes are regulated by the processes of fusion and fission. Additionally, mitochondria display high mobility in numerous cells, traveling through the cytosol via the cytoskeletal transport system. Consequently, mitochondrial dynamics play a pivotal role in maintaining mitochondrial quality [25]. Some researchers found that mitochondrial fusion was significantly increased in the tissues of patients with liver cancer, mitochondrial fusion was also inhibited in liver cancer cell lines after knockdown of *OPA1*, and the growth of liver cancer cells cultured *in vitro* and tumor formation in mice were inhibited [26]. In LUAD, the mitochondrial fusion process caused by *OPA1* is activated, enhancing mitochondrial metabolism to promote tumor growth and inhibit apoptosis. *OPA1* overexpression can also increase glycolytic activity to enable cancer cells in LUAD patients to achieve immune escape [27]. In addition, researchers found that upregulation of OPA1 expression was associated with poor prognosis in breast cancer, and the research team reduced proliferation, migration, and invasion of breast cancer cells *in vitro* and *in vivo* by inhibiting *OPA1* expression. Further research showed that *OPA1* silencing inhibited breast cancer cell growth and invasion by up-regulating the expression levels of the 148/152 miRNA family while not reducing mitochondrial respiration [28]. In pancreatic ductal adenocarcinoma (PDAC), the presence of cancer stem cells (CSCs) was associated with high invasiveness of PDAC, while OPA1 overexpression was found in CSCs and has a regulatory effect on tumorsphere formation [29]. In summary, we make a conjecture based on previous studies. The expression level of *OPA1* may play a very important role in the occurrence and development of pan-cancer.

Therefore, we will explore the level of *OPA1* gene expression in pan-cancer, its mutation types, and the relationship of these alterations with patient prognosis and immune microenvironment, as well as the molecular mechanisms that may be involved. This will provide a theoretical basis for further investigation of the possibility of using *OPA1* as a new prognostic marker for tumors.

## RESULTS

### Expression levels of *OPA1* in tumors and paraneoplastic tissues

**Figure 1:**
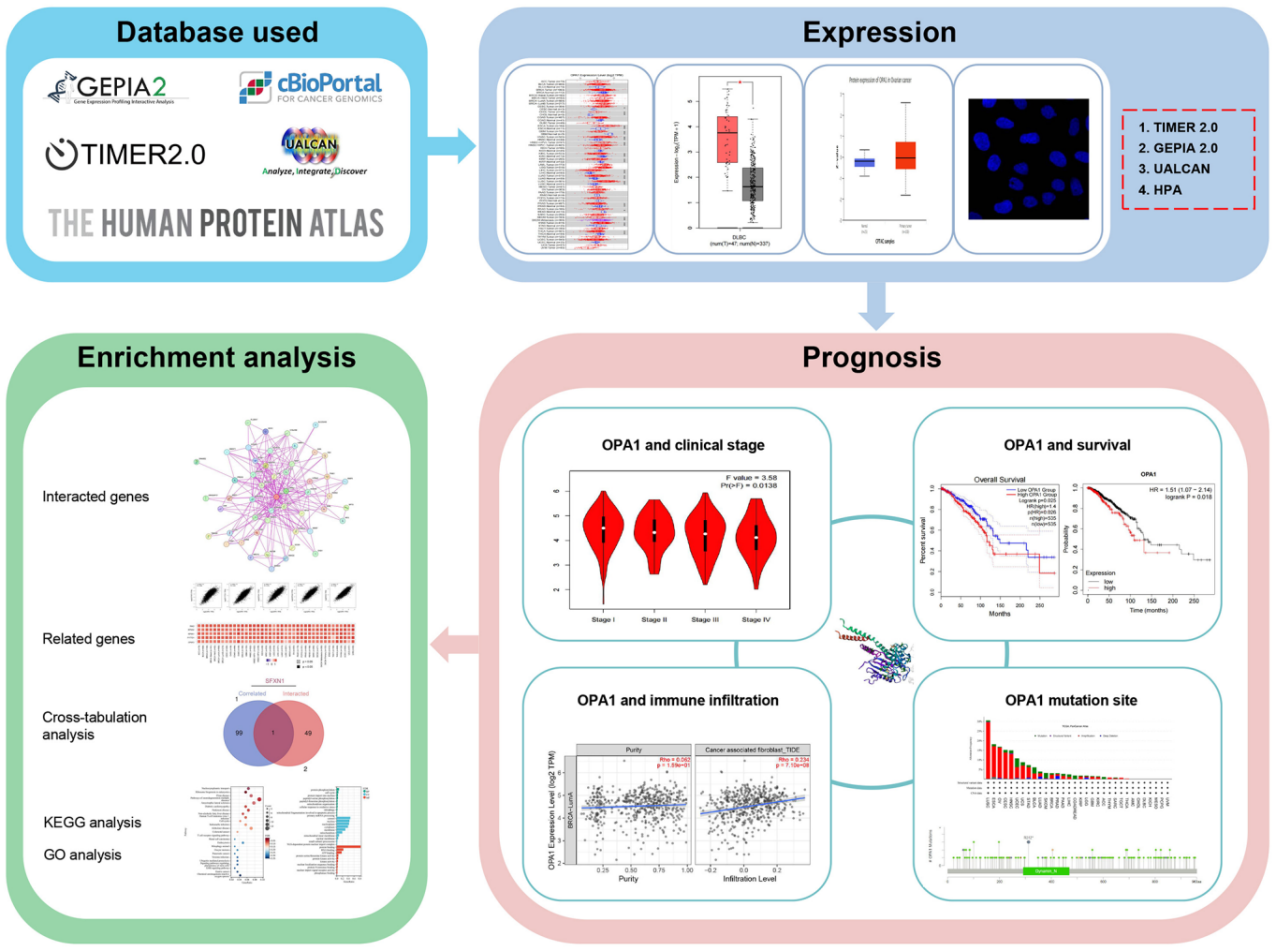
The flow chart of the whole study.

**Figure 2:**
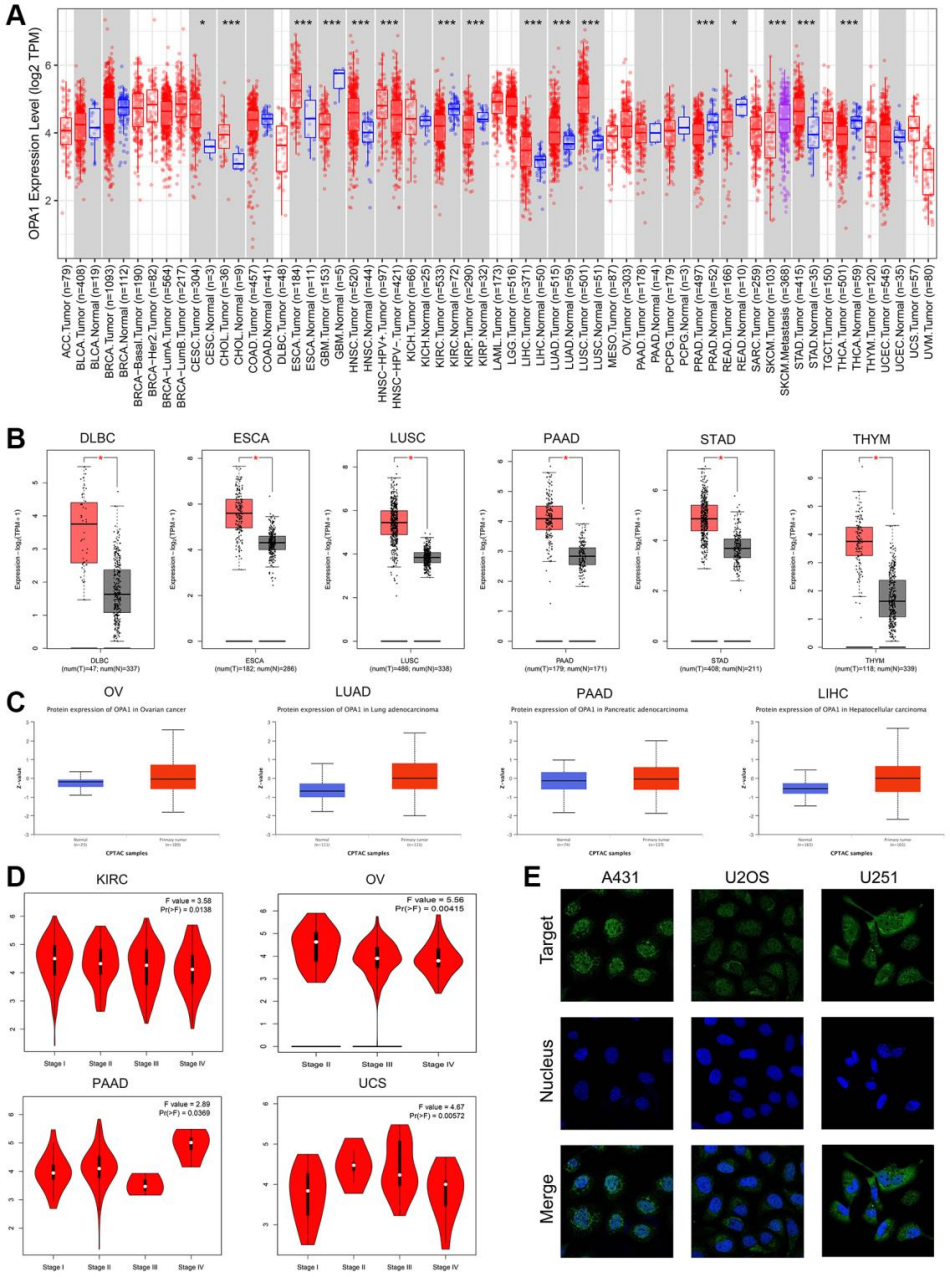
**The expression levels of *OPA1* in different tumors and normal samples.** (**A**) The expression levels of the *OPA1* gene in different tumors and paratumoral tissues were analyzed by TIMER2. ^*^p < 0.05, ^**^p < 0.01, ^***^p < 0.001. (**B**) The expression levels of the *OPA1* gene in DLBC, ESCA, LUSC, PAAD, STAD, THYM, and the normal samples, were analyzed by GEPIA. ^*^p < 0.05. (**C**) The protein expression of *OPA1* in OV, LUAD, PAAD, LIHC, and the normal samples was analyzed by UALCAN. (**D**) The expression level of *OPA1* in the different stages of KIRC, OV, PAAD, and UCS, was analyzed by GEPIA. (**E**) The immunofluorescence in osteosarcoma cell lines (A431, U2OS, and U251), was analyzed by HPA.

The workflow of our study is shown in Figure 1. We used the TIMER 2.0 database to compare *OPA1* expression levels in different tumors and normal tissues in the TCGA database, and the results were displayed in Figure 2A. The results showed that the expression levels of *OPA1* in CESC, CHOL, ESCA, GBM, HNSC, KIRC, KIRP, LIHC, LUAD, LUSC, and STAD were significantly higher than that of the corresponding normal tissues (Figure 2A).

Using the GEPIA database, we performed a joint analysis of *OPA1* expression data in various tumors from the TCGA and GTEx databases and found that *OPA1* was expressed at higher levels in DLBC, ESCA, LUSC, PAAD, STAD, and THYM than in control normal tissues, with significance (Figure 2B). By comparing *OPA1* expression at the protein level in different tumors and normal tissues using the UALCAN database, we found that *OPA1* protein expression levels were significantly elevated in OV, LUAD, PAAD, and LIHC compared to normal tissues (Figure 2C).

The results from the GEPIA2 database also demonstrated that *OPA1* expression levels were increased at different stages of KIRC, OV, PAAD, and UCS. (Figure 2D).

In addition, we used the HPA database to observe the subcellular localization of *OPA1*, and we observed by immunofluorescence imaging that the *OPA1* protein was localized both in the nucleus and on the nuclear membrane in A431, U2OS, and U251 cell lines. (Figure 2E).

### Prognostic value of *OPA1*

**Figure 3:**
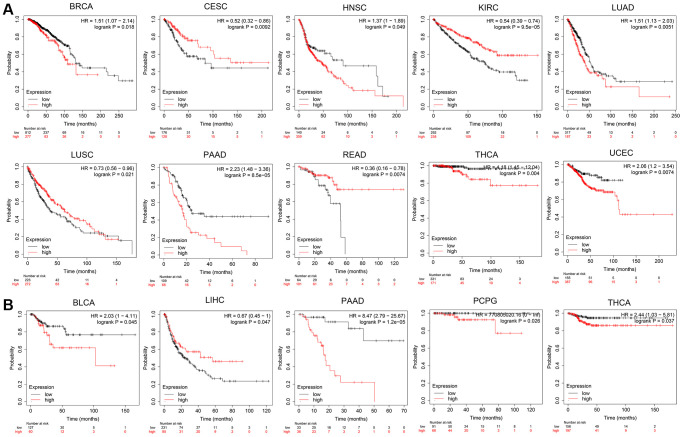
**The prognosis comparison of tumor patients in the *OPA1* high expression group and the low expression group, analyzed by Kaplan-Meier plotter.** (**A**) Overall survival and (**B**) relapse-free survival.

Based on the median expression level of *OPA1* in tumor cells of tumor patients, we divided the patients into high and low-expression groups. Then use the Kaplan-Meier plotter to analyze and visualize the survival of tumor patients with different *OPA1* expression levels at different periods. As shown in Figure 3A, patients with tumors including BRCA, HNSC, LUAD, PAAD, THCA, and UCEC, in the *OPA1* high expression group, had significantly lower overall survival rates than the patients with these tumors in the *OPA1* low expression group. However, the overall survival of CESC, KIRC, LUCS and READ patients in the high *OPA1* expression group was higher than that of the low expression group. For patients with BLCA, PAAD, PCPG, and THCA, relapse-free survival was also significantly lower in the *OPA1* high-expression group than in the low-expression group. However, patients with LIHC with high *OPA1* expression levels had significantly higher relapse-free survival rates than those with LIHC with low *OPA1* expression levels (Figure 3B).

**Figure 4:**
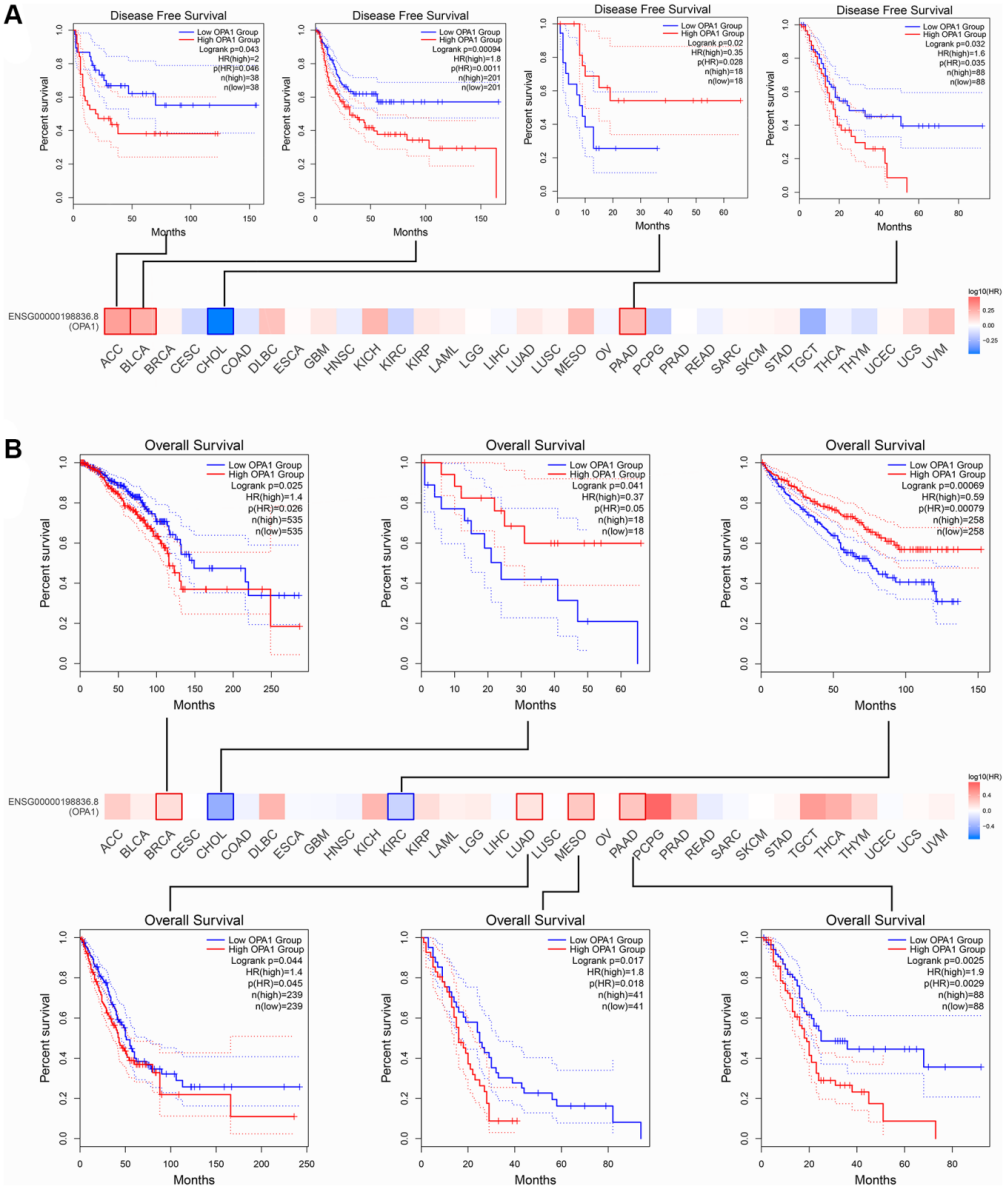
**The prognosis comparison of tumor patients in the *OPA1* high expression group and the low expression group, analyzed by GEPIA.** (**A**) Disease-free survival and (**B**) overall survival.

The results of the analysis through the GEPIA database were similar to those of Kaplan-Meier plotter. ACC, BLCA, and PAAD patients with low *OPA1* expression levels had higher disease-free survival than patients with high *OPA1* expression levels. However, CHOL patients with high *OPA1* expression levels had higher disease-free survival than those with low *OPA1* expression levels (Figure 4A). The results shown in Figure 4B indicated that the overall survival of BRCA, LUAD, MESO, and PAAD patients with high *OPA1* expression levels was lower than low-expression group patients. However, CHOL and KIRC patients in the high *OPA1* group had higher overall survival than the low *OPA1* group (Figure 4B).

### *OPA1* gene mutation site analysis

**Figure 5:**
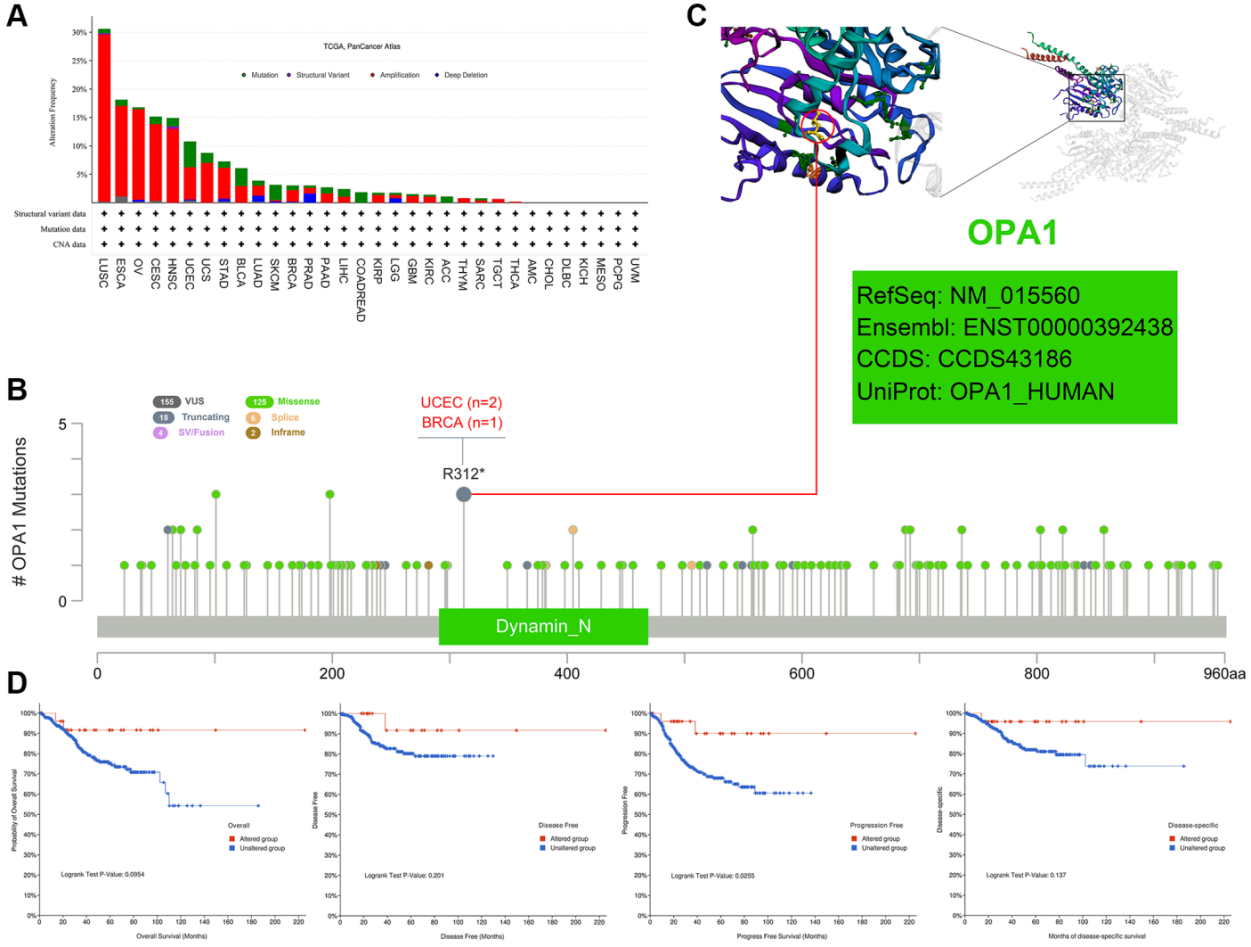
**Mutations of *OPA1* in individual tumors in the TCGA database, analyzed by cBioPortal.** (**A**) The mutation types of *OPA1* in individual tumors. (**B**) Mutation sites of *OPA1* in UCEC and BRCA. (**C**) 3D structure diagram of *OPA1* mutation site. (**D**) Relationship between *OPA1* mutations and survival in UCEC patients.

We analyzed the genetic alteration status of *OPA1* in different tumor samples from the TCGA cohort. As shown in Figure 5A, the highest frequency of *OPA1* alterations (29%) was found in patients with Lung Squamous cell carcinoma, where “amplification” was the predominant type. In addition, the amplification rate of *OPA1* in patients with ESCA, OV, CESC, and HNSC all exceeded 10%. Notably, the type of alterations in the *OPA1* gene in colorectal adenocarcinoma cases were all mutations (~2% frequency).

Figure 5B showed the type, locus, type of cases, and number of mutations in the *OPA1* gene. These mutations of unclear significance can neither be classified as favorable nor deleterious mutations and were referred to as VUS mutations. The most common of these were missense mutations. In two UCEC and one BRCA patient, both detected alterations in the R312^*^ site of the dynamin-N protein, which subsequently led to *OPA1* protein truncation. Figure 5C showed the R312^*^ site in the 3D structure of the *OPA1* protein. We also analyzed the impact of *OPA1* mutations on the prognosis of UCEC patients. The results showed that UCEC patients with *OPA1* mutations had a significantly higher PFS than UCEC patients without *OPA1* mutations (*P* < 0.05) (Figure 5D).

### Relationship between *OPA1* expression level and tumor immune microenvironment

**Figure 6:**
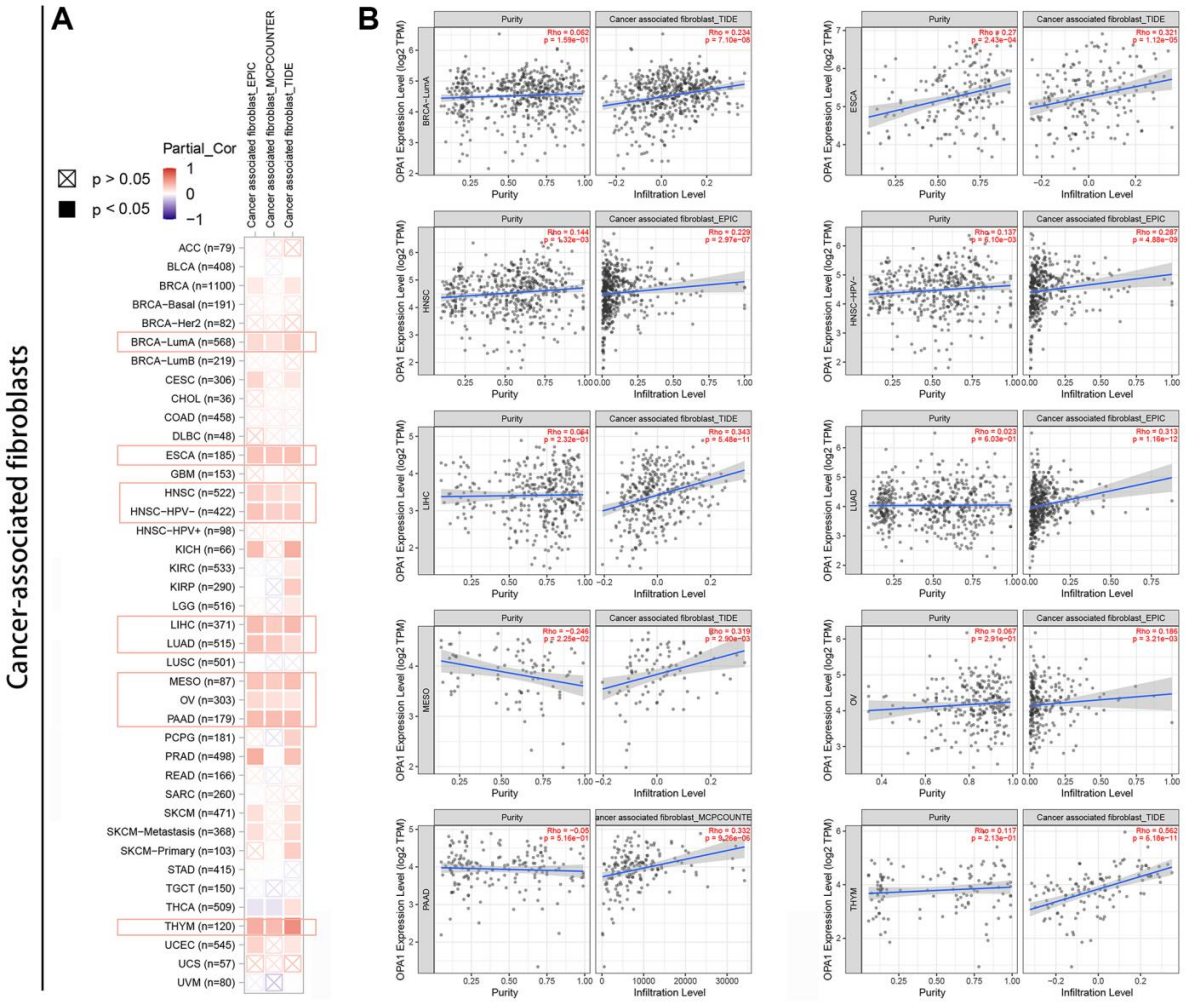
**Relationship between *OPA1* expression and cancer-associated fibroblast infiltration in pan-cancers**. (**A**) Heat map and (**B**) scatter plot.

Tumor filter cells are an important component of the tumor immune microenvironment and are closely associated with cancer development, progression, and metastasis [30, 31]. Here, we used the TIMER tool to analyze relevant data from the TCGA database to explore the statistical relationship between *OPA1* expression levels and the level of immune cell infiltration in pan-cancer. After processing the data using the EPIC, MCPCOUNTER, and TIDE algorithms, we found a statistically positive correlation between *OPA1* expression levels in BRCA, ESCA, HNSC, LIHC, LUAD, MESO, OV, PAAD and THYM and the estimated infiltration values of cancer-associated fibroblasts (Figure 6A, 6B).

### Enrichment analysis of *OPA1* and its related genes

**Figure 7:**
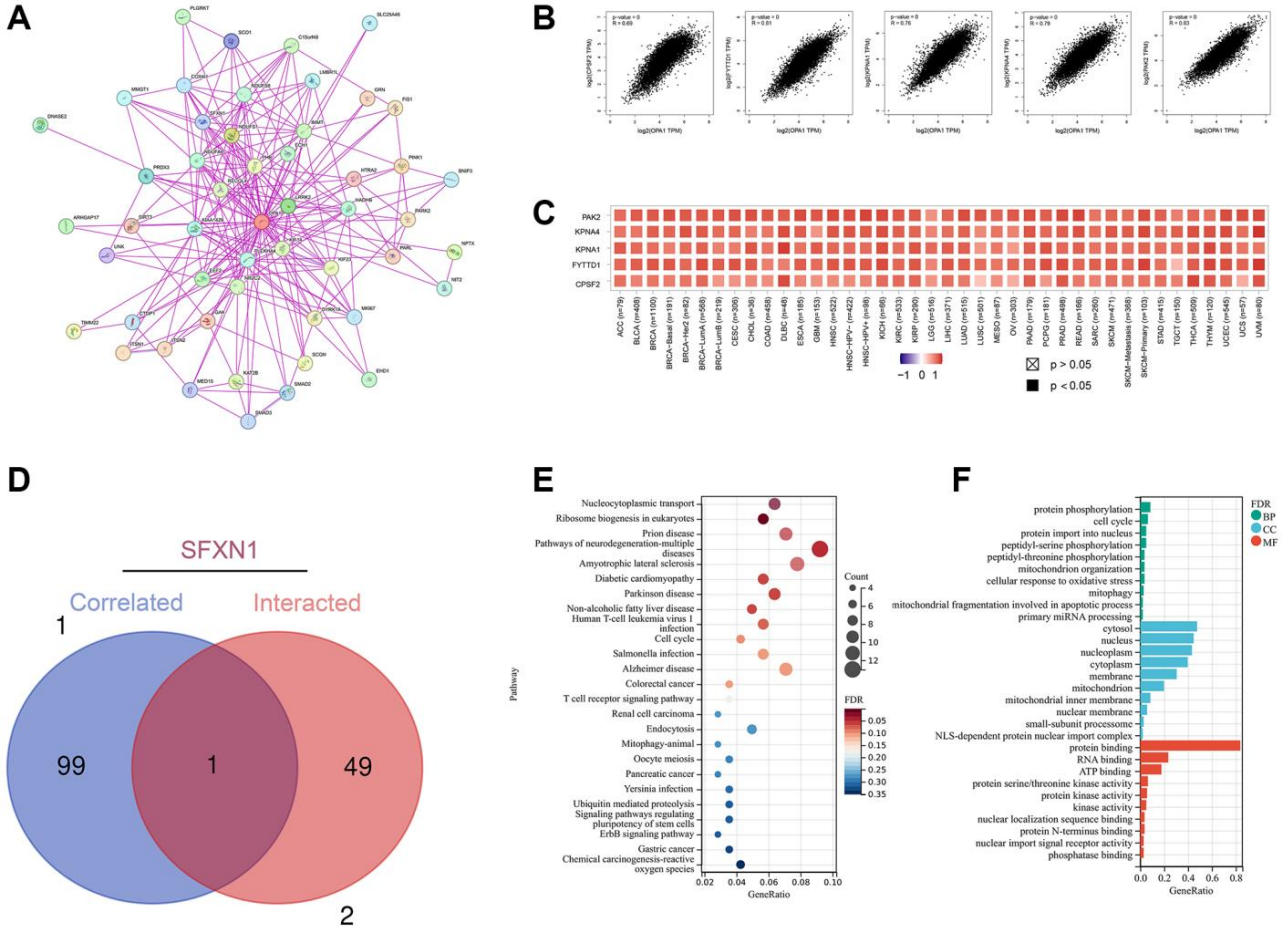
**Enrichment analysis of *OPA1*-related genes.** (**A**) *OPA1*-binding proteins analyzed by STRING. (**B**) Expression correlation between *OPA1* and the selected top 100 correlated genes including PAK2, KPNA4, KPNA1, FYTTD1, and CPSF2. (**C**) The corresponding heatmap of PAK2, KPNA4, KPNA1, FYTTD1, and CPSF2 in different cancer types. (**D**) The intersection analysis of genes corrected and interacted with *OPA1*. (**E**) KEGG pathway analysis of *OPA1* the genes correlated and interacted. (**F**) GO analysis of *OPA1* and the corrected and interacted genes.

To further elucidate the molecular mechanisms of *OPA1* gene involvement in tumor development, we screened genes targeting *OPA1*-binding proteins and genes associated with *OPA1* expression and performed enrichment analysis of these genes to identify the signaling pathways in which they function together. By using the STRING tool, we screened 50 proteins that bind to *OPA1*. Figure 7A shows the interaction network of these proteins. Next, we used the GEPIA2 tool combined with all tumor expression data of TCGA to obtain the top 100 genes associated with *OPA1* expression. As shown in Figure 7B, *OPA1* expression level was positively correlated with CPSF2 (R = 0.69), FYTTD1 (R = 0.81), KPNA1 (R = 0.76), KPNA4 (R = 0.76), and PAK2 (R = 0.83) genes. The heat map in Figure 7C showed a positive correlation between the expression levels of *OPA1* and the above five genes in pan-cancer. Cross-tabulation analysis of *OPA1*-related genes and interacting genes revealed a common member of these two groups of genes, SFXN1 (Figure 7D).

KEGG analysis revealed that *OPA1*-related genes may affect tumorigenesis and development mainly by affecting nucleocytoplasmic transport, ribosome biogenesis in eukaryotes, and pathways of neurodegeneration-multiple diseases (Figure 7E). GO analysis also showed that *OPA1*-related genes were associated with biological processes such as protein phosphorylation, protein import into the nucleus, protein binding, RNA binding, and others (Figure 7F).

## DISCUSSION

Our study indicates a significant presence or mutation of OPA1 in various tumors, demonstrating strong correlations with protein phosphorylation, patient prognosis, immune cell infiltration, and tumor mutational load. *OPA1* was found to play important roles in angiogenesis in the physiological state and tumors [18]. Angiogenesis is the formation of new blood vessel branches from old blood vessels, a process that is essential for wound healing and tumor growth [32]. Angiogenesis requires energy, which in human cells are provided by mitochondria, so some researchers have hypothesized that angiogenesis stimulates mitochondrial respiration [33]. However, new research suggests that mitochondrial functions were not limited to energy conversion, but were also involved in complex physiological processes such as cell differentiation, apoptosis, and immune signaling [34].

The function of mitochondria is closely related to their morphology [34]. Under normal physiological conditions, mitochondria perform their corresponding physiological functions by altering the balance of fusion and division [35]. Mitochondrial fusion proteins (MFN) 1 and 2 were located on the outer mitochondrial membrane (OMM) and were responsible for outer mitochondrial membrane fusion, and the optic nerve atrophy protein *OPA1* is located on the inner mitochondrial membrane (IMM) and is responsible for inner mitochondrial membrane fusion, and all three together regulate mitochondrial fusion. The cristae formed by the inner membrane invagination of mitochondria were essential for cellular metabolism, and *OPA1* plays a crucial role in maintaining the mitochondrial cristae structure [36]. *OPA1* may play a regulatory role during apoptosis by participating in cristae junction formation and maintenance [37]. During angiogenesis, *OPA1* can coordinate angiogenesis by affecting the activity of the NF-κB signaling pathway and the expression of angiogenic genes [18]. These studies suggest that *OPA1* plays important roles in both cell cycle regulation and tumor-related biological processes such as angiogenesis. However, our literature search revealed that there is no article on the pan-cancer analysis of *OPA1*, so a comprehensive analysis of *OPA1* in tumors is necessary. To this end, we analyzed *OPA1* expression, genetic alterations, patient survival prognosis, and immune infiltration in a total of 33 different tumor species based on an online database.

Previous studies have shown that mitochondria play a key role in tumor development, including providing energy for tumor cell growth, controlling redox, calcium homeostasis, transcriptional regulation, and controlling cell death. Metabolically, mitochondrial metabolites such as fumaric acid, succinic acid, and 2-hydroxyglutarate (2-HG) drive tumorigenesis, and mitochondria provide energy for anabolic processes in tumor cells [38]. Mitochondria also promote the transformation of normal cells into malignant cells through three main mechanisms: (1) Mitochondrial reactive oxygen species (ROS) promote the accumulation of potential carcinogenic DNA defects. (2) ROS plays an important role in activating potential carcinogenic signaling pathways [39]. (3) Functional defects in mitochondrial permeability transition are necessary for the survival of newly formed malignant tumor precursors [40, 41].

We found that *OPA1* was highly expressed in most tumors but had different prognostic effects in patients with different tumors. Patients with ACC, BRCA, HNSC, LUAD, MESO, PAAD, THCA, UCEC, BLCA, and PCPG in the *OPA1* high expression group had a worse prognosis, while *OPA1* high expression in CHOL, CESC, KIRC, READ, and LIHC patients had the opposite prognostic impact. Taking BRCA as an example, previous researchers have demonstrated through *in vivo* and *in vitro* experiments that knockdown of *OPA1* expression can reduce proliferation migration and invasion of breast cancer cells, and *OPA1* silencing can increase miRNA levels of the 148/152 family that inhibit tumor growth and invasion. Therefore, *OPA1* has been listed as a drug target for TNBC inhibition [28]. However, there is no conclusive evidence to explain why upregulation of *OPA1* expression is associated with better prognosis in CHOL, CESC, KIRC, READ, and LIHC patients. But combining our research with previous studies, we make the following inference: The reasons may include the following three aspects: First, each cancer has its unique mutation spectrum, *OPA1* and a variety of interacting genes play a biological function together, and in the *OPA1* interacting genes, the members that affect the development of different tumors are different. Secondly, the high expression of *OPA1* promotes mitochondrial function, which may also affect endocrine levels and different tissues have different responses to changes in hormone levels, such as the breast, prostate, testis, ovary, and other tissues are more sensitive to changes in hormone levels, and are more likely to cause cancer due to endocrine interference [21–23]. Thirdly, whether the high expression of *OPA1* plays a crucial role in the development of the above tumors also needs to be further verified by experiments. This is because the elevated expression of *OPA1* may simply be due to the alteration of normal tissues during the anti-tumor process. Fourth, the statistical difference may be due to insufficient sample size. Taking LIHC as an example, some researchers found that *OPA1* downregulation can enhance the sensitivity of hepatocellular carcinoma to chemotherapy drug sorafenib, which indicates that low expression of *OPA1* is associated with a better prognosis of hepatocellular carcinoma. Contrary to our results, we believe that this difference may be caused by insufficient sample size [42]. Therefore, although we have demonstrated that high expression of *OPA1* is associated with poor prognosis in a variety of tumors, specific analysis is needed for specific tumors.

In the present study, we investigated the mutation of the *OPA1* gene in tumors and its relationship with the prognosis of tumor patients. Mutations in *OPA1* were significantly associated with high survival rates in CESC. Through gene interaction analysis, we found that, *OPA1* expression level was positively correlated with CPSF2 (R = 0.69), FYTTD1 (R = 0.81), KPNA1 (R = 0.76), KPNA4 (R = 0.76), and PAK2 (R = 0.83) genes. Among them, PTC patients with high expression of CPSF2 had a higher risk of recurrence [43], high expression of KPNA-1 was associated with tamoxifen resistance and metastasis in breast cancer patients [44], KPNA-4 can be used as a biomarker for diagnosis and prognosis of hepatocellular carcinoma [45], and can also promote the metastasis of prostate cancer [46], and PAK2 can promote the migration and proliferation of salivary adenoid cystic carcinoma [47]. As for SFXN1, it is a mitochondrial serine transporter required for single-carbon metabolism, which plays an important role in the development of LUAD and can be used as an independent prognostic marker and therapeutic target for LUAD [48, 49]. There is currently no literature reporting a direct interaction between SFXN1 and OPA1. However, phosphorylation modifications, represented by serine residues, play a significant role in mitochondrial dynamics [50]. This could potentially serve as a mechanism for the interaction between SFXN1 and OPA1. Therefore, *OPA1* interacts with a variety of genes that affect tumor metastasis and prognosis. *OPA1* and these genes may work together in some signaling pathways and play roles in the occurrence and development of tumors.

In addition, we performed an enrichment analysis of *OPA1* and its related and interacting genes, and the results showed that these genes play important roles in related signaling pathways such as nucleoplasmic transport and ribosome biosynthesis, and may influence tumor pathogenesis by affecting protein binding, RNA binding, and ATP binding.

The immune microenvironment is also an important influencing factor in tumorigenesis and development. Previous studies have shown that cancer-associated fibroblasts (CAF) are inhibitory intermediates in the tumor microenvironment and are associated with poor prognosis. They can polarize the responsive immune population, including macrophages, by secreting immune regulatory factors. Our prognostic analysis results have shown that the expression level of *OPA1* is significantly correlated with the prognosis of cancer patients, so we speculate that *OPA1* may affect the prognosis of patients by affecting the infiltration level of cancer-associated fibroblasts. As we suspected, the results showed a significant correlation between high *OPA1* expression in tumors and high infiltration levels of cancer-associated fibroblasts.

## CONCLUSION

In our study, we analyzed the role of *OPA1* in the development of pancytopenia. Our findings suggest that *OPA1* is highly expressed or mutated in a variety of tumors and is strongly associated with protein phosphorylation, patient prognosis, immune cell infiltration, and tumor mutational load. These findings help us to better understand the role of *OPA1* in tumorigenesis development and potentially use it as a prognostic marker in some tumors.

## METHODS

### *OPA1* gene expression analysis

We used the TIMER 2.0 database to analyze *OPA1* expression levels in various tumors and normal tissues adjacent to tumors [51]. Then, *OPA1* expression level data from TCGA and GTEx databases were jointly analyzed using the GEPIA tool to obtain *OPA1* expression differences in cancer and normal tissues [52]. To explore the expression of *OPA1* at the protein level, we used the UALCAN database to mine the TCGA database for *OPA1* protein expression data [53]. Then, we analyzed *OPA1* gene expression level changes in various tumors at different stages through the GEPIA database. Taking the GEPIA database as an example, we can enter “OPA1” in the search box of GEPIA to search, then click the “DIY Expression” module, select “Boxplot” or “Stage plot”, and then click the tumor name to be analyzed in the “Datasets Selection” column to obtain the expression level of *OPA1* in various tumors and tumor stages.

### Survival analysis

To explore the association of *OPA1* with the prognosis of cancer, we used the Kaplan-Meier plotter and the GEPIA databases to analyze the relationship between *OPA1* expression levels and the OS and DFS of tumor patients and listed tumor types with significant statistical differences [54]. We entered “OPA1” in the search box of GEPIA to search, then clicked on the “Survival” module, selected “overall survival” or “RFS”, and then clicked on the tumor name to be analyzed in the “Datasets Selection” column to obtain the relationship between *OPA1* and various tumor prognosis. The Kaplan-Meier plotter database operates similarly.

### Analysis of *OPA1* gene mutations

The cBioPortal database was used to analyze mutations in *OPA1* in tumor tissue [55]. First, we found the ‘Quick selection’ section, then clicked on ‘TCGA Pan-Cancer Atlas Study’, and then typed ‘*OPA1*’ in the search field to query *OPA1* gene alterations. In the ‘Cancer Type Summary’ module, there is the TCGA database of *OPA1* alteration frequency, mutation type, and CNC in all tumors. By clicking on the “Mutation” module, we queried the information of the *OPA1* mutation site displayed as a 3D structure map. In the “Comparison” module, we compared the changes in OS, DFS, PFS, and DSS of cancer patients with and without *OPA1* gene alterations in the TCGA database, and the results were represented as Kaplan-Meier plots and indicated with log-rank *p*-values on the plots.

### *OPA1* subcellular localization

The Human Protein Atlas (HPA) database contains a wealth of human proteomics, transcriptomics, and systems biology data [56]. It contains a variety of protein expressions in various tumor tissues and normal tissues. Our study used the HPA database to analyze the localization of *OPA1* proteins in three tumor cell lines: A431, U2OS, and U251.

### Correlation analysis of *OPA1* with tumor-associated fibroblast infiltration

To investigate how *OPA1* expression in tumor cells affects the level of immune cell infiltration in the tumor microenvironment, we used the “Immunogene” module of the TIMER 2.0 tool to perform an analysis of *OPA1* expression and infiltration of cancer-associated fibroblasts in the TCGA database. The immune cells we selected were cancer-related fibroblasts. We used three algorithms, TIDE, MCPCOUNTER, and EPIC, to assess the level of cancer-associated fibroblast and plotted these data as heat and scatter plots.

### Enrichment analysis of *OPA1* and the related genes

We first opened the STRING database, entered ‘*OPA1*’ in the ‘Protein name’ field, and selected ‘Homo sapiens’ in the ‘Organism’ field [57]. Subsequently, the parameters were set as follows: In the ‘Minimum required interaction score’ field, select ‘Low confidence (0.150)’. In the ‘Meaning of network edges’ field, select ‘Evidence’, in the ‘Maximum number of interactors shown’ field, select ‘No more than 50 interactors’, and in the first shell and ‘Active interaction source’, select ‘Experiment’. Finally, click “Update” to obtain the experimentally determined proteins bound to *OPA1*. In addition, we used the “Expression Analysis-Similar Gene Detection” tool in GEPIA2 to extract the top 100 genes most associated with *OPA1* in the TCGA tumor and normal tissue database. We performed coupled gene Pearson correlation analysis of *OPA1* and selected genes using the “Expression Analysis - Correlation Analysis” module. The data of these genes were then plotted as a heat map using the “Exploration - Gene_Corr” module of TIMER 2.0.

In order to cross-tabulate the top 100 genes that bind to *OPA1* with the top 50 interacting genes, we used the Jvenn database [58]. Then we uploaded the list of all these genes to the DAVID [58] database and set them up as follows: identifier selection (“OFFICIAL_GENE_SYMBOL”), species selection (“Homo sapiens”). In addition, we performed KEGG and GO analysis of the above gene list using the “Functional Annotation Map” module of DAVID. The analyzed data were downloaded and those with *p*-values less than 0.05 were selected for the next step of processing.

### Tools and websites

cBioPortal: https://www.cBioPortal.org/; DAVID: https://david.ncifcrf.gov/; GEPIA: http://gepia.cancer-pku.cn/; GEPIA2: http://gepia2.cancer-pku.cn/#index; HPA: https://www.proteinatlas.org/; Venn: http://bioinformatics.psb.ugent.be/webtools/Venn/; Kaplan-Meier plotter: http://kmplot.com/analysis/; STRING: https://cn.string-db.org/; TIMER 2.0: http://timer.cistrome.org/; UALCAN: http://ualcan.path.uab.edu/index.html.

### Availability of data and materials

All data generated or analyzed during this study are included in this published article.
